# The “Labeled” Side of COVID-19 in India: Psychosocial Perspectives on Islamophobia During the Pandemic

**DOI:** 10.3389/fpsyt.2020.604949

**Published:** 2021-01-22

**Authors:** Kanika K. Ahuja, Debanjan Banerjee

**Affiliations:** ^1^Department of Psychology, Lady Shri Ram College for Women, New Delhi, India; ^2^Department of Psychiatry, National Institute of Mental Health and Neurosciences (NIMHANS), Bengaluru, India

**Keywords:** COVID-19, coronavirus, pandemic, India, xenophobia, islamophobia

## Abstract

The Coronavirus disease 2019 (COVID-19) has emerged as a global public health threat over the last few months. Historically, infectious disease outbreaks like the plague, Influenza, cholera, HIV, etc. have generated stigma, prejudice, “othering” and xenophobia, against certain communities. One such prevalent form of xenophobia, is Islamophobia or “fear and discrimination against the Muslims.” Though debated over its various facets and definitions, it is on the rise worldwide. India, being a socio-politically diverse and populous nation, has been facing unique challenges during COVID-19. Considering Hinduism and Islam are the two major religious communities, the subcontinent has witnessed complex dynamics in their relationship throughout history. The pandemic has further instigated Islamophobia, and consequent discrimination, as well as unrest. This can have significant effect of public behavior and health. In the recent past, few legislations in India were interpreted to be Islamophobic and generated nation-wide protest, which provided a fertile backdrop against the discriminative effects of the pandemic. Keeping this in background, this commentary highlights the social contexts of increase in Islamophobia in India during the pandemic, discusses the possible psychological explanations and public health impact, as well as outlines some ways to mitigate it focusing on collectivism.

## Introduction

The world has faced a new public health threat over the last few months. The Coronavirus disease 2019 (COVID-19) has affected nearly 69 million and claimed the lives of more than 1.57 million, around the world (World Health Organization COVID-19 Situation Report, as on December 10, 2020). As the major focus of management gets shifted to exploring the biological cure for the virus, the social effects get largely neglected. Pandemics of such large-scale are not merely biological phenomena; they also have socio-economic and psychological offshoots that can outlast the infection itself. Efforts to address epidemics have historically been hindered by the failure to take into account the psychosocial aspects of these diseases. Besides the direct psychological impact of the infection secondary to the uncertainty and fear of an unknown infection, the social implications can be immense. Mass panic, agitation, competition for access to health care and discrimination based on social classes, religion or ethnicities are common (1). This leads to societal stigma and certain minority populations being targeted amidst the already enhanced crisis. It has been seen that earlier epidemics such as Ebola exacerbated structural inequities, such as gender (2) and prejudice toward outgroup members (3). Biological disasters have been shown to influence human behavior, increasing illogical, irrational, provocative, polarized and aggressive behavior (4). Misinformation and misinterpretation of social media can further compound these reactions. From the very time COVID-19 was declared to be a pandemic, it has been labeled as “Kung Flu” or “Chinese virus,” thus sparking international tensions and blame (5). Every nation has been facing their unique psychosocial challenges and India, being one of the socio-culturally and religion-wise diverse and populous country, specific issues have been faced by certain “outgroups.” One such outgroup in the Indian context are the Muslims, who have time and gain come under accusations and harassment related to the infection. Keeping this in background, this article provides some perspectives for rising Islamophobia in India, in the context of COVID-19 pandemic and briefly draws implications for policy and practice. The authors would like to provide an open disclaimer for their neutral viewpoints in this commentary as mental health professionals, and not endorsing any specific political ideology. Being researchers in the field of mental health, the authors would like to emphasize on their neutral stance and aim to critically analyze the psychosocial and cultural contexts during the pandemic based on factual data rather than opine about any particular ideas, religion or governance.

## Islamophobia and India: Situational Contexts Preceding COVID-19

Hinduism and Islam are the two most prevalent religions in India. Hindus and Muslims have had a complex co-existence (6), at times characterized by violent conflicts, such as the partition of the country in 1947, 1989 Kashmir violence, 2002 Gujarat riots, and 2013 Muzaffarnagar riots. The relationship, however, has not always been fraught with violence. In this socio-culturally dynamic and secular sub-continent, both these religious groups have also harmoniously co-habited most of the times, mutually respecting each other's rituals and traditions. Islamophobia or “fear and suspicion” against Muslims though rising in legal literature, has been a matter of major debate and discussion. It has been defined in various ways based on the varied schools of thought, and typified as private, structural and dialectic Islamophobia (7). Irrespective of the different aspects to the “fear,” the concerns for Islamophobia have always been on the civil liberties and human right threats posed to a particular religious community. Legally, usage of this term has been debated on similar lines as of the term “homophobia,” to denote the “fear” against any community or groups to be more irrational and aberrant, rather than structural and strategic (8). Islamophobia has reached record highs in some Western countries in the recent past (9). Sirgy et al. (10) showed an inverse relationship between subjective well-being and islamophobia (considering it as a type of xenophobia). Similarly, the National Well-Being Index in South Africa was reported to have a significant relationship with attitudes toward immigrants (11). Such prejudiced attitudes against certain “communities” amplified during crisis situations can fuel social chaos, stigma, agitation, mutual blame and panic that in turn predispose to psychological stress and trauma. Social justice and welfare might be impaired because of these attributes (12).

The situation in India in the preceding months of the COVID-19 infection has, however, been contextually significant. The Citizenship Amendment Act (CAA) was enacted by the Indian Government on December 12, 2019. The Act amends the Indian citizenship to illegal migrants from the neighboring countries, who entered India before 2014, subsequent to the religious prosecutions. It does not, however, mention about the Muslim communities, who had fled from these countries due to the same reason. The amendment was widely criticized as discrimination against Muslims, and protests broke out rapidly across the country, though the agenda and intentions of the protestors were widely heterogenous (13). The associated proposal of the National Register of Citizens (NRC) further added to the agitation. This sparked concerns among the Indian Muslims and people of lower socio-economic classes if they would be denied citizenship and rendered stateless (14). The state forms a major part of “collective identity” and consequently, when protests were already widespread related to the perceived loss of political rights, culture and land, the pandemic outbreak simply snowballed the ongoing communal turmoil. Pandemics have been historically politicized and laws implemented accordingly, like at times of HIV in Africa or Influenza in Europe (15). This time, certain legislations and the resultant public reaction provided a fertile ground for the genesis of existent xenophobia, with the virus acting as the catalyst.

In fact, the site of Shaheen Bagh, one of the major foci of anti-CAA protests, was cleared as late as March 24, 2020, when the number of confirmed coronavirus cases stood at 564 (14). As mentioned before, during the initiation of COVID-19 pandemic in India, the communal atmosphere was tense. Rising anti-Islamic rhetoric, hate crimes, violation of human rights, and mutual blame have been on the rise in context of the protests mentioned above (13). In this background of communal strife, it is not surprising that the stage was already set for Islamophobia, fear, hatred of, or prejudice against the Muslims in general. All India needed was a trigger, which was unfortunately provided by an infection like COVID-19.

When COVID-19 started spreading in India, and Delhi, in particular, some media reports started describing the outbreak in Delhi as the “Tablighi spread.” On March 31, a police complaint was lodged against seven people, including the emir of the Tablighi Jamaat (who was subsequently booked on April 15 for culpable homicide not amounting to murder by Delhi Police) for holding a gathering of over 3,000 members at its global headquarters in Nizamuddin. This gathering allegedly violated orders against large gatherings and social distancing norms put in place to contain the spread of COVID-19. These members traveled to different states from Delhi after attending the congregation, became the carriers of the virus, infecting hundreds. There were comments mentioning that 30% of all COVID-19 cases in the country, 1,023 of 2,902 reported at the time, were linked to this event.

It has been contended that even though other faith communities hosted similar large-scale gatherings, events held by Muslim associations such as the Tablighi Jamaat were scapegoated (16). While the Tablighi Jamaat congregated between 13 and 15 March, temples like Siddhivinayak and Mahakaleshwar closed on March 16; Shirdi Saibaba Mandir and Shani Shingnapur Temple closed on 17 March; Vaishno Devi on 18 March, and the Kashi Vishwanath Temple was operating until 20 March—a day after the Government had urged the public for “social distancing.” Similarly, places of worship pertaining to other religious faiths also hosted community events during this time period. The Tablighi Jamaat meeting in Delhi being singled as the main vector of the coronavirus, led to a significant increase in anti-Islamic sentiments, including boycotts of businesses of those from the Muslim community, separation of patients based on their religion, refusal to admit Muslims, resulting in the alleged deaths of two newborn babies after their mothers were denied admission, randomly quarantining Muslims, and subjecting Muslim healthcare and essential workers to violence and harassment (17).

People were asked not to buy vegetables from Muslims, a video of which went viral. Controversial posters also showed up on fruit shops in Jamshedpur to demarcate the faith of the shopkeepers [(18); www.indiatoday.in, April 27, 2020]. Other fake videos showing how the Muslim missionary group were spitting or coughing on others to spread corona deliberately too became viral. There were also reports that alleged that the Tablighi Jamaat members admitted to a hospital refused to take medicines, spit on their hands and touched staircase railing, and misbehaved with the medical staff (19). Terms such as “Coronajihad” became popular on social media, as an expression of willful misuse of the COVID-19 infection by certain religious communities, in order to establish their superiority. Also termed as “Talibani crime” or “Corona Terrorism,” these quotes fueled the fire of Islamophobia and further strained the inter-religious relationships. Since March 28, tweets with the hashtag #CoronaJihad have appeared nearly 3,00,000 times and potentially seen by 165 million people on Twitter, according to the data shared by Equality Labs, a digital human rights group (20). Though the authenticity of the statistics is debatable, such pejorative terms can easily provoke the ongoing political tensions and lead to law-and-order situations, during pandemics.

Iyer and Chakravarty (21) analyzed the media reportage from March 20 to April 27, 2020 using an open-source media analysis platform Media Cloud. 11,074 stories were published from 271 media sources with the term “Tablighi Jamaat” during the period, of which 94 per cent were English stories that appeared in the print media. At its peak, on April 2, Media Cloud tracked as many as 1,451 news articles covering the Tablighi Jamaat case. 1.5-10 per cent of the stories had words with negative connotations such as “violating,” “crime,” “spitting,” “terrorist,” and “jihad.” These stories fed into an epidemic of Islamophobic fake news and hate speech. Some fact checking websites such as Media Scanner exposed over a 100 instances of Islamophobic misinformation during this period. Aggravated by the atmosphere of fear and uncertainty during the pandemic, it is not surprising that such media narratives demonized the entire Muslim community. Research [e.g., (12)] found the non-Muslim population to indeed report negative attitudes toward the Muslims, which interestingly reduced their own well-being. Participants, especially those who were older, were more likely to believe that the outbreak of COVID-19 in India was primarily due to Muslims. Such incidents, in fact, led the World Health Organization (WHO) to caution against profiling cases based on racial, religious and ethnic lines for the greater good of the community (22, 23).

Anxieties over the coronavirus thus merged with longstanding Islamophobia in India. Infectious diseases are well-known to invoke widespread fear. History shows that such fear can be used to legitimize discrimination and violence against certain segments of the society. Indeed, blaming “the other” is a way to make mysterious and distressing diseases somewhat understandable, and hopefully even controllable (24). “Othering” is a concept, originally having philosophical connotations, which tends to create the “we vs. they” dichotomy, thus attempting to alienate certain “others” from the self and in broader terms, the center of the society (25). It has eventually emerged into a term in social science that encompasses multiple expressions of prejudice based on xenophobic identities. Examples of this are numerous. “Othering” and consequent prejudice have been commonly seen against the peasants in the classical Bubonic plague of the thirteenth Century, the Indians during the Asiatic Cholera at times of the British rule, against the Chinese in Severe Acute Respiratory Syndrome (SARS) outbreak, as racism during Ebola infection and finally against the same-sex men during the Human Immunodeficiency Virus (HIV) upsurge, which has even been labeled as the “Gay Plague” (26).

The notion of “othering” has also been amply explored by Indian writers such as Guru and Nandy. Guru (27), writing on the marginalization and ghettoization of the Dalits uses Ambedkar's conception of the Indian nation. Ambedkar argued that India comprises of two nations: Puruskrut *Bharat* (privileged) that represents the twice-born castes who are spatially, socially, and culturally different from the *Bahiskrut Bharat* (under-privileged), the untouchables, helping to comprehend the claim for social equality that sustains spatial practices of exclusion. Nandy (28), in the context of Hindu-Muslim relations, asserts that religious fundamentalism and religious violence are not merely by-products of, but the burden of modernization and Westernization. He insists that “traditional India” is inherently adaptive and tolerant (p. 79) and most instances of communal violence are the work of people motivated by “entirely secular, political cost-calculations” [(29), p. 72]. Rather than striving to become idealized global citizens who shed all prejudices and perceived differences, Asians living in diverse communities should learn to accept the “otherness of others.” In the context of Islamophobia, socio-cultural “othering” has unleashed common processes and conditions that propagate religion-based inequality and marginality.

## COVID-19 and Islamophobia: Psychosocial Perspectives

Various psychological factors play a role behind rise in Islamophobia as a result of the COVID-19 outbreak. Some of them are discussed below:

### Disease Avoidance Model

This proposes that stigmatization of various groups might result either directly or indirectly from an evolved predisposition to avoid diseased conspecifics (30). Such stigmatization includes emotional and cognitive components. The former directly activates disgust and contamination, such as when non-Muslims feel anger and disgust toward Muslims leading to motivation to avoid them; and the latter whereby the label of COVID-19 brings to mind associations with Muslims, irrespective of their accuracy, indirectly activating disgust and contamination. This model contends that psychological mechanisms have evolved to protect people against the threat of infectious diseases. While such disease avoidance has adaptive utility, it results in an overgeneralized prejudice toward people who are perceived to be potential carriers of disease (31).

This model was tested by Huang et al. (32) in their study conducted during the height of the H1N1 swine-flu epidemic. They reported that even temporary exposure to a disease-related threat, by making participants read a passage about the swine- flu epidemic, was associated with increased anti-immigrant prejudice. Interestingly, people who were vaccinated and therefore felt protected from disease, reported less prejudice than do people who are not vaccinated. They also found that simple interventions like having some participants wash their hands significantly influenced participants' perceptions of out-group members.

Faulkner et al. (33), on similar lines, found that chronic and temporarily aroused feelings of vulnerability to disease contributed to negative attitudes toward foreign (but not familiar) immigrants. They, however, did not find significant xenophobic tendencies toward outgroups who were subjectively familiar. Extending this further, it is possible that Islamophobic attitudes in India could be currently held by those who did not have much interaction with this religious group in the first place. Culturally discordant beliefs and religious ideas might give rise to discomfort and consequent hostility. It seems clear that perceived vulnerability to infectious diseases moderates prejudice toward the “out” group. This has significant implications for both policy and practice.

### The Pathogen Prevalence Hypothesis

This is another theory which suggests that people living in regions with a high prevalence of pathogens show increased collectivistic behaviors. This leads to greater conformity and higher xenophobia (34). Research has indeed provided evidence of a positive association between country-wide measures of pathogen prevalence, collectivism and xenophobia (35). Cashdan and Steele (36) also substantiated a relationship between disease prevalence and collectivist values—especially those values pertaining to adherence to group norms. It seems that people do respond to perceived vulnerability by becoming more collectivistic. It has been contended that as many disease-causing pathogens are invisible, and their actions mysterious, adhering to ritualized behavioral practices has historically reduced the risk of infection (37). Individuals who fail to conform to these behavioral traditions, on one hand, pose a health threat to self and others. On the other hand, a collective behavioral tendency toward obedience and conformity can lead to disease-specific benefits, such as mitigating the spread of disease (by maintaining one's distance from the outgroup).

Kim et al. (3) also tested the influence of individualism and collectivism on xenophobic response to the threat of Ebola. They found that those who perceived themselves to be more vulnerable to Ebola were more xenophobic and displayed greater prejudice toward West Africans and immigrants, although this association was weaker among people who were more collectivistic. Perhaps, the more individualism is rising in urban spaces in India, the more the fear of COVID-19 is leading to Islamophobia. This highlights an important gap that researchers should move quickly to fill in the coming weeks and months, to manage the impact of the pandemic. The conceptualization of xenophobia (Islamophobia in the current context) needs to be considered as an important component of public health and psychological preparedness for the post-pandemic aftermath.

### Terror Management Theory

This provides yet another angle to understand xenophobia. The terror management theory (38) posits that awareness of the inevitability of death exerts a significant influence on various aspects of human emotion, thought, motivation, and behavior. The uncertainty and possibility of death evokes strong fear in people. Applying the terror management theory, it may be postulated that the costs associated with failing to detect a contagious individual (e.g., getting infected yourself, or even death) outweigh the costs of misidentifying a healthy person as a disease carrier. As a result, disease-avoidance mechanisms occasionally act out at targets who are not legitimate sources of disease (31). Individuals stand to gain by keeping away from those social groups, whom they perceive as carriers of the disease, Muslims in the current context. This acts as a psychological defense of feeling “safe” and “assured” at the face of a crisis, by attributing the onus of the problem to the “other.” Social attribution theories support this model, as attributing an external locus of control to an unprecedented disaster, not only decreases the uncertainty but also helps in “misperceived sense of assurance” (39).

Other cases of racially oriented stigmatization have been noted in earlier outbreaks. For instance, in 1993, when an outbreak of an unexplained pulmonary illness occurred in the southwestern United States, the term “Navajo disease” was used in reference to the patient zero, a Novajo woman. Even after the specific hantavirus that caused this outbreak was isolated, the term “Navajo disease” continued to be used, ignoring the fact that non-Navajos were also becoming ill. This led to fears of disease coupled with anti-Indian racism (40).

Fear of death also led to disproportionate stigmatization in the 1994 plague outbreak in Surat, India. Within a week after the infection had been identified publicly, half a million people fled Surat, many of whom were turned away from neighboring communities and cities. Trains and International flights to India were canceled, outgoing passengers were quarantined (including Mother Teresa). The stigmatization was clearly disproportionate to the extent of the outbreak, resulting in severe economic losses and major health and social problems (41). The potent effect of stigmatization was seen yet again during the SARS epidemic. The perceived linkage between SARS and ethnicity led to the irrational avoidance of Asians (especially Chinese) in many parts of the world. Many countries imposed excessively stringent restrictions on travelers from Asia (42).

### Shift Toward Conservatism and Dogmatism

This proposition explains hatred toward other religious communities at times of crises. Previous research has shown that uncertainty, fear of death, instability of social systems, and the potential to evoke disgust promotes socially conservative attitudes (43). In fact, people in the United States (U.S.) were found to report more conservative attitudes after the terrorist attacks of 9/11/01 than before, regardless of whether they identified themselves as liberal vs. conservative (44). Similarly, the Ebola epidemic in 2014 was found to influence voter behavior in two psychologically distinct ways: increased inclination to vote for politically conservative candidates and increased inclination to conform to popular opinion (45). While research on the socio-political aspects of COVID-19 is still upcoming, an investigation (46) assessed political ideology, gender role conformity, and gender stereotypes among 695 U.S. adults before (2 months preceding) vs. during the pandemic. Their findings suggested that the pandemic promoted preference for traditional gender roles.

It is believed that adopting a conservative ideology enables individuals to manage feelings of threat and anxiety that environmental uncertainty evokes (47). All of us caught in the midst of this pandemic not only face uncertainty but also the threat of contracting the COVID-19 virus from our surrounding social and physical environments. This leads to people getting primed with an exponentially growing pathogen threat—a prime that is likely to activate disgust to motivate pathogen avoidance. And considering that Muslims in the present context represented pathogen threat, the feelings of disgust and motivation to avoid them became a natural by-product, in a population that was already showing signs of Islamophobia. As people become more conservative, they might show greater signs of prejudice toward the out-group.

### The Way Forward: Implications for Policy and Practice

Stigma is much more than just a negative outcome of a certain disease or a pandemic; it is an illness in itself, comorbid with respect to its marked physical conditions (41). A large body of research has shown being the target of discrimination causes both psychological and physical suffering [e.g., (48, 49)]. To discuss the Indian scenario, the case fatality rate of COVID-19 is lower than many other countries. However, given its ethno-religious diversity, political polarizations, influence of social media and above all, the mere population surge, there are more worrisome issues than just the statistics of infection (50). The new forms of Islamophobia in India in the wake of COVID-19 could have far-reaching political, social, and health implications. The increased alienation of the Muslim community could increase the rates of infections and mortality, as infected individuals would be too afraid to come forward due to fear of being attacked (51). It could also lead to ghettoization of Muslims, where they feel safe to live in overcrowded places with “their own,” but would find it difficult to practice social distancing. Further, Islamophobia in the minds of the majority community could promote vigilante violence, resulting in deepening religious fault lines. Communalism and Islamophobia can provoke mass agitation, violence, panic, non-compliance to precautionary measures and public chaos, all of which can be detrimental for the overall well-being, especially when the viral cases are rising each day.

Understanding how to improve prejudicial attitudes while promoting other social benefits is therefore of critical importance. Paradoxically, denying the existence of negative attitudes only deepens divisions. The hiding and mistrust of health care workers and police officials among the Muslim community derives from stigma, fear, and lack of information. The more COVID-19 is stigmatized, the more it will aggravate marginalization of these communities, and divide the country along religious lines. Media, especially social media, plays an important part to play in such situations. In a digital era, when COVID-19 is literally an “infodemic,” content related to conspiracy theories and graphic yet selective video content from some anecdotal events turn viral (52). At times of pandemics, the need for information rises and people due to their anxiety tend to consume any form of news, irrespective of their authenticity. During the lockdown, the internet usage in India has tripled and thus the surge of misinformation (53). All that it takes, is one senseless forward to wrong hands, to snowball the fear and stigma. There is also a pressing need to assess the impact of repeated media consumption around COVID-19. Media across the world has reported misleading and discriminatory information which can cause deterioration in mental health and well-being. It may be argued that, labels like “Wuhan Virus” or “Chinese virus pandemonium” linking COVID-19 to a race outside or “Tablighi outbreak” in Delhi are divisive, inflammatory and counterproductive (5). It will result in unrest and further brew feelings of Islamophobia. Positive reports in media such as members of the Tablighi Jammat, perceived to be responsible for the spread of COVID, later volunteered to donate blood for plasma therapy into COVID-19 patients should be highlighted. Other positive stories of how Muslims celebrated their festival Eid with self-restrictions, and practiced social distancing need to be highlighted. In fact, social media is also a uniquely powerful tool to promote social awareness against discrimination by health education about the virus and information-education-communication (IEC) activities for communal harmony.

Since Islamophobia is associated with increased vulnerability of disease, the Government has already taken proactive steps toward assuring public to mitigate their fears. In April, 2020 it had issued an advisory to address the social stigma associated with the pandemic, urging people not to label any community or area for the spread of the virus. The advisory also aptly highlighted how fear and anxiety caused by infectious disease outbreaks can lead to social isolation, stigma and marginalization of certain communities (54). Fear of COVID-19 begets prejudice. If the threat of the contagion can be eliminated, the mental responses associated with disease-related threats will follow suit. The legal and administrative authorities have tried their best to contain the infection, maintain the regulations of lockdown and preserve public harmony (55). However, sometimes the perceived importance of comments related to religious content might need to be monitored, which can easily trigger human sentiments. Allaying the anxiety due to the pandemic itself can be a good starting point. Perhaps the low mortality rates of COVID-19, despite its highly infectious nature, could be highlighted. People's fears could also be reduced by reinforcing preventive measures that lower the risk of contracting COVID-19, such as repetitive hand washing, precautions when buying essentials, wearing of masks in a proper manner, continued social distancing, etc. Healthy hygiene habits will have to be incorporated into our new normal for a long time. This will lead to increased perceptions of protection from disease, thereby not only reducing prejudice toward people who are not legitimate carriers of disease, but also enhance well-being. Authentic sources of information from the official websites of the World Health Organization (WHO), Center for Disease Prevention and Control (CDC), Indian Council of Medical Research (ICMR) and the Ministry of Health and Family Welfare (MoHFw), Government of India (GOI) can be harnessed for awareness. Assessment and understanding of the sentiments at grass-root levels are necessary to prevent Islamophobia. Certain religious minorities might feel cornered in general, that gets amplified by crisis, which can be mitigated by promoting their social inclusion and integration (56). Social security and belongingness go a long way in assuring people and decreasing their agitation. The Indian Pandemic Act of 1897 needs a total overhaul, in the context of COVID-19 and legal provisions and penalties for any form of religious discrimination or fake news related to the same, especially during such disasters, need to be incorporated.

## Conclusion

India is gradually pacing up in the rate of COVID-19 infection. As cases rise daily, the virus does not discriminate between social classes, race, ethnicity or religion. Every potential case and fatality are a concern for the nation's public health, all the more due to the disproportionate population compared to the available resources. It is ironic that civilization has always dealt with a disaster better in medical, than in social terms. Xenophobia increases individualism and hate in some, whereas perceived stigma, loneliness and polarization in others (57). Both, can lead to stress, add to the burden of psychological disorders and post-traumatic stress, for years to come. Once again, the authors would like to reiterate their neutral stance and lack of affiliation to any particular religious schools or ideologies. The pandemic will eventually be over, but the phobic attitudes toward certain religious communities might get strengthened. For any nation, especially India which has been declared secular by its Constitution, such Islamophobic sentiments can harm the community at a large, irrespective of class or religion (58). The Government has tried its best to preserve harmony and solidarity, especially during the ongoing and trying times. Understanding and appreciating the socio-cultural contexts of islamophobia through the psychological lens will only refine it for a more collectivistic approach, that is a necessity during a global threat such as COVID-19. Religions have co-existed and supported each other mutually for decades, and that has added to the remarkable resilience of the subcontinent against various other threats in past. Humanity is in this crisis together, and inter-group solidarity can foster positivism and hope, that can boost psychological well-being, as well as immunity. COVID-19 gives the nation yet another chance to dwell deep into the problem of Islamophobia and adopt options to mitigate it.

## Data Availability Statement

The original contributions presented in the study are included in the article/supplementary material, further inquiries can be directed to the corresponding author/s.

## Author Contributions

All authors have contributed equally to the planning, conceptualizing, literature review, drafting, editing, read and approved the final version of the manuscript.

## Conflict of Interest

The authors declare that the research was conducted in the absence of any commercial or financial relationships that could be construed as a potential conflict of interest.
